# Differentiation of Malignant and Benign Incidental Breast Lesions Detected by Chest Multidetector-Row Computed Tomography: Added Value of Quantitative Enhancement Analysis

**DOI:** 10.1371/journal.pone.0154569

**Published:** 2016-04-29

**Authors:** Yu-Pang Lin, Hsian-He Hsu, Kai-Hsiung Ko, Chi-Ming Chu, Yu-Ching Chou, Wei-Chou Chang, Tsun-Hou Chang

**Affiliations:** 1 Department of Radiology, National Defense Medical Center, Taipei, Taiwan; 2 Department of Radiology, Tri-Service General Hospital, Taipei, Taiwan; 3 School of Public Health, National Defense Medical Center, Taipei, Taiwan; Geisel School of Medicine at Dartmouth College, UNITED STATES

## Abstract

To retrospectively determine the association between breast lesion morphology and malignancy and to determine the optimal value of lesion enhancement (HU, Hounsfield units) to improve the diagnostic accuracy of breast cancer in patients with incidental breast lesions (IBLs). A total of 97 patients with 102 IBLs detected from July 2009 to December 2012 were enrolled in this study. Two radiologists analyzed CT images for the presence of malignancy based on the morphology of the lesions alone and in combination with an enhancement value (HU) analysis. There were 36 malignant and 66 benign IBLs. When the morphology and enhancement values were combined, the sensitivity, specificity, and accuracy were 92%, 97%, and 95%, respectively, for reader 1 and 89%, 94%, and 92%, respectively, for reader 2. The addition of HU values led to correct changes in the diagnosis; specifically, the accuracy of the diagnosis of reader 1 and reader 2 improved by 6.9% and 11.8%, respectively. The addition of the enhancement value (HU) to the CT morphology improved the diagnostic accuracy in the differentiation of malignant from benign IBLs by using the region of interest (ROI) to measure the HU within the most suspicious part of the lesion.

## Introduction

Multidetector-row computed tomography (MDCT) is increasingly being used in the diagnosis and surveillance of chest disease. Occasionally, incidental breast lesions (IBLs) are encountered during a MDCT examination [1–7]. Computed tomography (CT) has been shown to have high diagnostic efficacy in the evaluation of breast tumours. In CT scans of known breast tumours, the lesion morphology and enhancement pattern can be used to differentiate benign from malignant lesions [8–13]. Moreover, investigators in previous studies explored the mean CT values (HU, Hounsfield units) of the lesions in patients with suspected breast cancer [14]. For example, Lindfors and Prionas et al. aimed to quantify the enhancement value of lesions identified by dedicated breast CT [15, 16]. Although the importance of morphology and the CT enhancement pattern in differentiating breast tumours has been reported in the previous literature, there has been no study focusing on the relationship between the enhancement values (HU) and malignancy of IBLs.

The aims of the present study were two-fold: (1) to determine the association between lesion morphology and breast cancer and (2) to determine the optimal value of lesion enhancement (HU) to improve the diagnostic accuracy of breast cancer in patients with IBLs.

## Materials and Methods

### Patients and lesions

This retrospective study was approved by the institutional review board of Tri-Service General Hospital. Informed consent for the diagnostic procedure was waived. A total of 13,651 patients, 123 of whom were identified to have IBLs, underwent contrast-enhanced chest CT scans at our hospital from July 2009 to December 2012. Of those 123 patients, 26 were excluded due to a history of breast cancer and/or insufficient diagnostic information. Finally, a total of 97 female patients were included in this retrospective study (mean age: 55.3 years, range: 27–91 years).

Of the 97 patients, the indications for chest CT included staging or a follow-up study for other malignancies (n = 21; non-small cell lung cancer, thyroid cancer, cervical cancer, gastric cancer, oesophageal cancer, renal cell carcinoma), benign neoplasms (n = 4; goitre, thymoma), abnormal chest radiographs (n = 35), CT pulmonary angiography (n = 14) and other pathologies (n = 23) (Table 1). Ninety-three patients had one breast lesion, three patients had two lesions, and one patient had three lesions. Therefore, a total of 102 IBLs were analyzed in our study.

**Table 1:** Indications of CT Scans which Detect Incidental Breast Lesions in 97 Patients. *NSCLC* non-small cell lung cancer, *COPD* chronic obstructive pulmonary disease.

Indications			n
Staging of other neoplasms			
	Malignant		
		NSCLC	11
		Thyroid cancer	3
		Cervical cancer	3
		Gastric cancer	2
		Esophageal cancer	1
		Renal cell carcinoma	1
	Benign		
		Goiter	2
		Thymoma	2
Abnormalities on chest x-ray film			
	Nodule, patch		15
	COPD		11
	Pleural effusion		9
CT pulmonary angiography			14
Other pathologies			23
Total			97

### CT technique

The patients underwent a 64-detector row CT (Brilliance, Philips Medical Systems, Cleveland, Ohio) scan of the chest in the supine position. The standard protocols in our hospital include unenhanced MDCT from the lung apices through the adrenal glands with the following parameters: slice thickness of 2.5 mm, reconstruction interval of 2.5 mm, rotation speed of 0.75 sec, pitch of 1.05–1.25, and 120 kVp; the effective tube current × time product ranged from 150–200 mAs. Subsequently, contrast-enhanced MDCT was performed with intravenous administration of 100 mL Iopromide (Ultravist-300, Schering, Berlin, Germany) or Iohexol (Omnipaque 300, General Electric Health Care, Princeton, NJ) using a mechanical power injector at a rate of 2.0–3.0 mL/sec. The scanning was executed 50 sec after the injection of the nonionic contrast medium with technical parameters identical to those used for unenhanced CT. For patients suspected to have an aortic dissection or pulmonary embolism, the contrast medium was administered at a rate of 3.0–4.0 mL/sec. The scanning was triggered after a bolus of contrast medium reached the descending aorta or pulmonary artery. The images were obtained by employing a standard soft-tissue algorithm (window width, 350 HU; level, 40 HU) and a retrospective lung algorithm (window width, 1000 HU; level, -700 HU).

### CT imaging interpretation

To assess the benefits of adding the CT enhancement value (HU) in the differentiation of malignancy from benign lesions, the diagnostic performance of morphology analysis alone was compared with that of morphology combined with CT enhancement analysis (both axial and coronal images). An enhancing breast lesion meant that the lesion had higher attenuation compared with the normal breast glandular tissue on contrast-enhanced CT. For the evaluation of the CT morphology alone, the CT images were retrospectively reviewed by two radiologists (H.H.H. and Y.P.L., with 25 and 3 years of experience, respectively). The radiologists were blinded to the imaging reports, clinical history, and final pathology of the lesion, and they independently interpreted the findings of the 102 IBLs. In cases of discordance, the rating was discussed until an agreement was reached. The results of the independent rating and consensus rating were all documented for the analysis. Due to the lack of a formal lexicon for CT breast imaging, a consensus analysis of morphology was achieved by using the Breast Imaging and Reporting Data System (Bi-RADS) lexicons of MR imaging-detected breast lesions as a reference [17].

**Fig 1:**
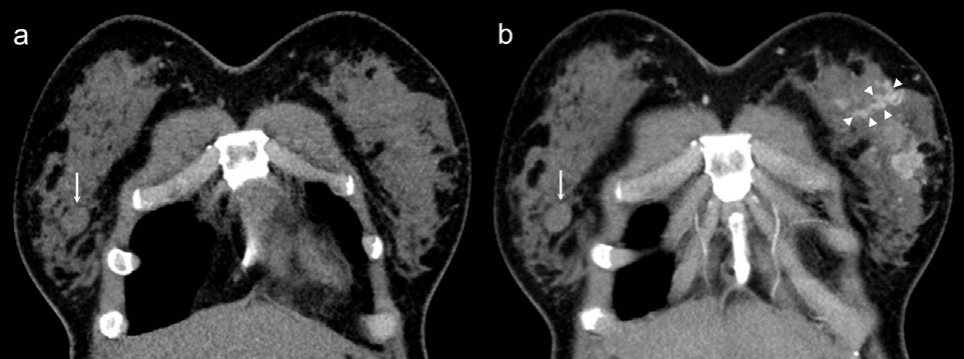
A 27-year-old female underwent chest CT for chronic cough. Coronal nonenhanced (a) and contrast-enhanced (b) CT scans showed an ovoid, well-defined fibroadenoma (arrow, 41 HU in nonenhanced CT and 47 HU in contrast-enhanced CT,ΔHU = 3) in right breast and multifocal nonmass enhancement (arrowheads, 39 HU in nonenhanced CT and 119 HU in contrast-enhanced CT, ΔHU = 77) in left breast. Histopathlogy revealed diagnosis of invasive ductal carcinoma.

The CT characteristics of each lesion were analyzed as follows: size or extent (greatest axial diameter), lesion type (mass or non-mass), shape (oval, round, lobulated, irregular), margin (circumscribed, indistinct, spiculated), the presence of calcifications or abnormal axillary nodes, and lesion conspicuity (visual enhancement). The non-mass lesions, also known as non-mass enhancement (focal, linear, ductal, segmental, and regional), indicated that the lesion appeared as an area of enhancement without an associated mass (Fig 1), as defined in the BI-RADS MR lexicon [17]. The diameter of a mass, as a measure of its size, was assessed on axial images with electronic callipers. The size of a non-mass lesion was delineated as the extent of enhancement. The axillary nodes were considered to be abnormal if they were larger than 1 cm in the maximum transverse dimension, and if there was presence of a lobulated cortical margin or absence of a fatty hilum. With regard to the visual enhancement of the breast lesions, contrast-enhanced CT images were evaluated first. If the lesion could not be identified, the unenhanced CT images were examined, and with the added information from that evaluation, the contrast-enhanced CT images were interpreted. The visual enhancement was evaluated by the two radiologists as a qualitative assessment, where ‘good’ indicated a more conspicuous lesion on enhanced CT images compared with unenhanced CT images; otherwise, the visual enhancement was described as ‘poor’.

To evaluate the combination of the CT imaging morphology with a quantitative enhancement analysis, the same radiologists who executed the first evaluation again decided whether each lesion was benign or malignant. To prevent recall bias, the evaluation of the combined CT imaging morphology and lesion enhancement (HU) was carried out six weeks after the evaluation of the CT morphology alone and in a different order from that of the first evaluation. Both readers were blinded to the initial imaging reports, clinical history, and final lesion pathology, and they independently analyzed the quantitative value of the enhancement (HU) of the IBLs. The enhancement values (HU) were assessed by placing a manually drawn region of interest (ROI) in the most suspicious part of the lesion on contrast-enhanced images. Sites of calcification were carefully avoided because they had relatively high HU values. To correctly draw the ROIs in the lesion, we set the contrast-enhanced image and a corresponding non-contrast image side-by-side to ensure that the images were at the same level. All ROIs were drawn as large as possible within the lesion. We used the adipose tissue density to normalize the lesion density (HU) and account for variations between image acquisitions. The lesion enhancement (ΔHU) was calculated as the difference between the normalized lesion density in the pre- and post-contrast images. The data were evaluated using the following equation: ΔHU = (HU post L-HU post A)- (HU pre L_-_ HU pre A) [16], where L and A represent the lesion attenuation and adipose tissue attenuation, respectively, measured in the pre-contrast (pre) and post-contrast (post) image sets.

### Statistical analysis

We entered the data into a computerized spreadsheet (Excel, Microsoft, Redmond, WA, USA). All analyses were performed using a commercially available software program (SPSS, version 19.0, SPSS Inc., Chicago, Illinois). The categorical variables related to malignancy were calculated by theχ^2^ test, and the odds ratios (ORs) with 95% confidence intervals (CIs) were determined. Moreover, the predictive abilities of the variables were compared using an area under the curve (AUC) analysis [18]. Statistically significant variables associated with malignancy identified in the univariate analysis (*P* < 0.05) were chosen for inclusion in a multivariate analysis using multiple logistic regression models to distinguish variables of independent statistical significance. The HU cut-off values in malignant breast lesions were determined using receiver operating characteristic (ROC) curve analysis. We used the kappa values to assess the interobserver agreement between the two readers regarding the decision of whether the lesion was benign or malignant. A kappa value of 0.00–0.20 indicated slight agreement; 0.21–0.40, fair agreement; 0.41–0.60, moderate agreement; 0.61–0.80, substantial agreement; and 0.81–1.00, almost perfect agreement [19]. Sensitivity and specificity were compared by McNemar statistics.

## Results

### Histological findings

A summary of the diagnoses of the IBLs is shown in Table 2. Thirty-six of these lesions were subsequently proven to be malignant, and 66 lesions were considered to be benign. For the 36 malignant lesions, all were proven to be malignant by biopsy and pathological diagnosis and included invasive ductal carcinoma (n = 23), invasive lobular carcinoma (n = 7), and ductal carcinoma *in situ* (n = 6). The 66 benign lesions were further followed by sonography or CT and were confirmed to have a satisfactorily benign appearance. The follow-up period was 1.5 years (n = 5), three years (n = 32), or five years (n = 29).

**Table 2:** Diagnosis of the 102 Incidental Breast Lesions.

Diagnosis		n
Malignant		36
	Invasive ductal carcinoma	23
	Invasive lobular carcinoma	7
	Ductal carcinoma in situ	6
Benign		66
	Fibroadenoma	57
	Fibrocystic change	3
	Cyst	2
	Breast abscess	1
	Intramammary lymph node	1
	Intraductal papilloma	1
	Scar	1
Total		102

### Associations between malignancy and imaging characteristics

The patient and lesion variables associated with malignancy are listed in Table 3. The lesion size, type, shape, margins, and presence of abnormal axillary lymph nodes predicted malignancy (*P* < 0.05 for each). Based on the area under the ROC curve (AUC), greater discrimination was provided by the shape (AUC = 0.84) and margin (AUC = 0.90). The age of the patient and presence of calcification were not significantly associated with malignancy.

**Table 3:** Rates of Benign and Malignant Lesions for 102 incidental Breast Lesions by Patient and CT Imaging Parameters. Note.—Unless otherwise indicated, data are numbers of lesions, with percentages in parentheses.

		All lesions	Benign	Malignant	Odds ratios		Chi-squares		Area under the curve
Parameters		No.	No. (%)	No. (%)	(95% CIs)		(*P* value)		(95% CIs)
		n = 102	n = 66	n = 36					
Age of the patient							0.22	(0.641)	0.48 (0.36, 0.60)
	< 50 y	45	28 (62)	17 (38)	1.0				
	≧ 50 y	57	38 (67)	19 (33)	0.8	(0.4–1.9)			
Lesion size/extent^a^							6.7	(0.009)	0.60 (0.49, 0.72)
	< 1cm	40	32 (80)	8(20)	1.0				
	≧ 1cm	62	34 (55)	28 (45)	3.3	(1.3–8.3)			
Lesion type							30.0	(<0.001)	0.72 (0.60,0.84)
	Mass	83	64 (77)	19 (23)	1.0				
	Non-mass	19	2 (11)	17 (89)	28	(6.0–137)			
Shape^b^							21.7	(<0.001)	0.84 (0.75, 0.93)
	Oval, round	51	48 (94)	3 (6)	1.0				
	Irregular, lobulated	32	16 (50)	16 (50)	16	(4.1–62.1)			
Margin^b^							36.0	(<0.001)	0.90 (0.81, 0.99)
	Circumscribed	64	59 (92)	5 (8)	1.0				
	Non-circumscribed	19	5 (26)	14 (74)	33	(8.4–130)			
With Calcification							0.12	(0.725)	0.53 (0.41, 0.64)
	No	73	48 (66)	25 (34)	1.0				
	Yes	29	18 (62)	11 (38)	1.2	(0.5–2.9)			
Axillary abnormal node							18.0	(<0.001)	0.65 (0.53, 0.77)
	No	88	64 (73)	24 (27)	1.0				
	Yes	14	2 (14)	12 (86)	16	(3.3–76.8)			
Visual Enhancement Pattern							6.5	(0.011)	0.65 (0.55, 0.76)
	Poor	30	25 (83)	5 (17)	1.0				
	Good	72	41 (57)	31 (43)	3.8	(1.3–10.9)			

^a^ Size for 83 mass lesions and extent for 19 non-mass lesions.

^b^ Shape and margin characteristics were analyzed in 83 mass lesions. *CIs* confidence intervals.

### The usefulness of CT in differentiating benign and malignant lesions

**Fig 2:**
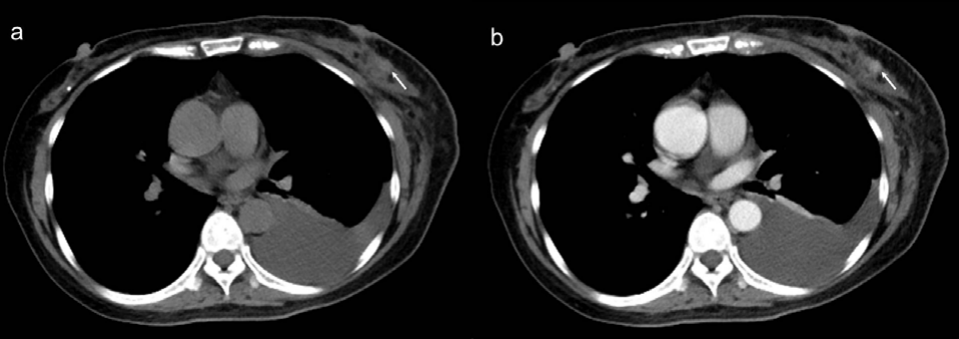
A 58-year-old female underwent chest CT for left pleural effusion. Axial nonenhanced (a) and contrast-enhanced (b) CT scans showed an irregular enhancing lesion (arrow, 35 HU in nonenhanced CT and 79 HU in contrast-enhanced CT, ΔHU = 40) with indistinct margin in left breast. Histopathlogy revealed diagnosis of invasive ductal carcinoma. Fluid cytology of the pleural effusion was proven to be malignant.

The cut-off CT values for differentiating benign and malignant IBLs and the AUCs are shown in Table 4. The differential enhancement between benign (14.1 HU ± 1.9, standard error) and malignant (46.8 HU ± 3.3) lesions was significant (*P* < 0.001) (Fig 1). The following results are clinically applicable and showed high diagnostic accuracy. The values useful for differentiating malignant from benign lesions are a value of more than 57 HU on contrast-enhanced CT images (sensitivity, 100%; specificity, 83%; accuracy, 89%) and a difference between the normalized lesion density on pre- and post-contrast CT images (ΔHU) of more than 33 HU (sensitivity, 83%; specificity, 95%; accuracy, 93%) (Fig 2); the ROC curve analyses yielded AUCs of 0.92 (95% CI, 0.87–0.98) and 0.93 (95% CI, 0.87–0.98), respectively, for these values. Based on the AUC values, the greatest discrimination was provided by the ΔHU.

**Table 4:** CT Cutoff Values for Differentiating Benign and Malignant Incidental Breast Lesions with Receiver Operating Characteristic (ROC) Curve Analysis. Note.—Numbers in parentheses are raw data; numbers in brackets are 95% confidence intervals. Percentages were rounded. *CIs* confidence intervals, *AUC* area under the receiver operating characteristic curve, *HU* Hounsfield units.

Parameters	Cutoff-Value	Sensitivity (%) [95% CIs]	Specificity (%) [95% CIs]	Accuracy (%)	AUC [95% CIs]
Pre-contrast CT values (HU)	32	72 (26/36) [54.8, 85.8]^c^	71 (47/66) [58.8, 81.7]	72 (73/102)	0.717 [0.611, 0.823]
Post-contrast enhanced CT values (HU)	57	100 (36/36) [90.2, 100]	83 (55/66) [72.1, 91.4]	89 (91/102)	0.917 [0.861, 0.973]
Difference between normalized lesion	33	83 (32/36) [73.9, 96.8]	95 (63/66) [87.3, 99.0]	93 (95/102)	0.922 [0.855, 0.988]
density in the pre-and post-contrast images (nd M					

Based on the multivariate analysis results shown in Table 5, the relationships of the margin and ΔHU between malignant and benign lesions were significant (*P* = 0.008 and *P* < 0.001). The lesion size and shape did not differ significantly according to the multiple logistic regression analysis.

**Table 5:** Risk Factors Associated with Malignancy: Univariate and Multivariate Results. Note.—Numbers in parentheses are number of malignant lesions out of total number of lesions.

Parameters^a^		Malignancy rate %		Univariate analysis		Multivariate analysis	
				OR (95% CIs)	*P*	OR (95% CIs)	*P*
Lesion size				3.8 (1.1–12.5)	0.032	1.1 (1.1–9.8)	0.94
	<1cm	11	(4/36)				
	≧1cm	32	(15/47)				
Shape				16 (4.1–62.1)	<0.001	3.7 (0.4–32.6)	0.242
	Oval, round	6	(3/51)				
	Irregular, lobulated	50	(16/32)				
Margin				33 (8.4–130)	<0.001	25.5 (2.3–283)	0.008
	Circumscribed	8	(5/64)				
	Non-circumscribed	74	(14/19)				
Difference between normalized							
leison density in the pre-and				76.3 (15.4–378)	<0.001	79.1 (7.5–838)	<0.001
post-contrast images (re-a							
	<33	6	(4/65)				
	≧33	83	(15/18)				

^a^ Nineteen non-mass lesions are excluded. *OR (95% CIs)* odds ratio (95% confidence intervals), *HU* Hounsfield units.

The sensitivity, specificity, and accuracy for the discrimination of malignant breast lesions by the CT morphology alone and by the combination of morphology and the difference between the normalized lesion density in pre- and post-contrast CT images (ΔHU) are listed in Table 6. When the CT morphology was used alone, the sensitivity, specificity, and accuracy of reader 2 (72%, 85%, 80%) were lower than those of reader 1 (81%, 92%, 88%). With the additional information provided by the quantitative ΔHU, the accuracy was improved for both reader 1 (added value = 6.9%, seven of 102 lesions) and reader 2 (added value = 11.8%, twelve of 102 lesions). In our study, there were specific improvements in the diagnostic accuracy of the readers, including the reader with less experience, following the addition of CT values. The sensitivity and specificity of reader 2 was significantly different (*P* = 0.014 and *P* = 0.031). The kappa values showed substantial to almost perfect agreement (k = 0.696 when diagnosed by morphology alone; k = 0.935 when diagnosed using morphology in combination with the ΔHU) in whether IBLs were malignant or benign based on CT imaging.

**Table 6:** Diagnostic Performance in the Differentiation of Malignant from Benign Incidental Breast Lesions. Note.—Numbers in parentheses are raw data. Percentages were rounded.

Parameter	Morphology alone		Morphology in combination with Δor^a^	
	Reader 1	Reader 2	Reader 1	Reader 2
Sensitivity(%)	81 (29/36)	72 (26/36)	92 (33/36)	89 (32/36)
Specificity(%)	92 (61/66)	85 (56/66)	97 (64/66)	94 (62/66)
Accuracy(%)	88 (90/102)	80 (82/102)	95 (97/102)	92 (94/102)

^a^ΔHU = difference between normalized lesion density in the pre-and post-contrast images.

## Discussion

Our study showed that contrast-enhanced breast CT offers a promising quantitative technique with which to predict malignancy in IBLs identified with routine chest CT. The quantitative findings suggested that there is improved specificity for contrast-enhanced chest CT in the detection of malignant IBLs. The diagnostic accuracy was improved by adding a quantitative enhancement analysis to the assessment of qualitative morphology features, including improved accuracy for a less-experienced radiologist.

Our results also support the findings of other investigators indicating that lesion morphology (qualitative features) is valuable from a diagnostic standpoint. In our study, the morphology analysis of the IBLs revealed that the features with the highest positive predictive value (PPV) for malignancy are non-circumscribed margins (PPV, 74%) and an irregular or lobulated shape (PPV, 50%). These findings are consistent with the previous literature [5, 9]. In addition, we found low PPV values for malignancy with features such as an oval or round shape (PPV, 6%) and circumscribed margins (PPV, 8%) (Table 3). These findings are also consistent with the results reported by Moyle et al. [5] and Inoue et al. [9]. The chi-square test revealed a significant relationship between the margins or shape of the lesion and malignancy (*P* < 0.001). In general, our data are concordant with the previous literature in concluding that non-circumscribed margins and an irregular or lobulated shape are more likely to be associated with malignancy.

**Fig 3:**
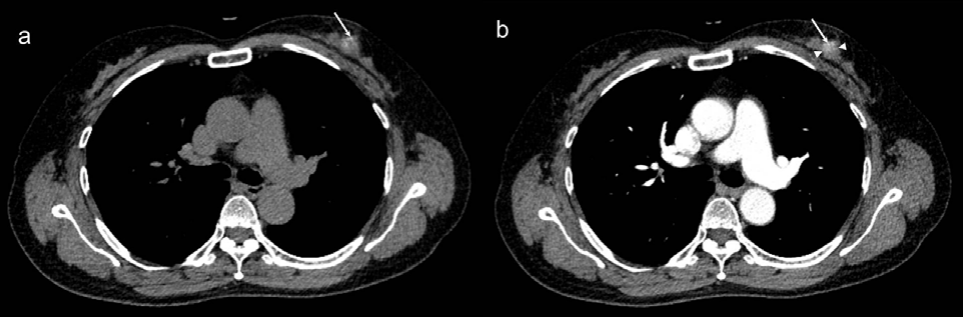
A 70-year-old female underwent chest CT for health examination. Axial nonenhanced (a) and contrast-enhanced (b) CT scans showed tiny calcifications (arrow) in left breast. The area adjacent to the calcifications showed enhancement (arrowheads, 82 HU in nonenhanced CT and 116 HU in contrast-enhanced CT, ΔHU = 34). Histopathlogy revealed diagnosis of ductal carcinoma in situ.

In the present study, malignant lesions were enhanced approximately 38.9 HU more than benign lesions [16]. In our study, the mean difference was 32.7 HU. Although the HU value is affected by various factors, such as the machine used, type of contrast medium, presence of calcification, and imaging acquisition time, these cut-off values can be of practical value for guiding judgement at an institution [14, 16]. Based on the AUC, excellent discrimination was achieved when ΔHU≥33 (AUC = 0.922) (Table 4). Additionally, the IBLs that had an HU value >57 on contrast-enhanced CT had a higher rate of malignancy (AUC = 0.917). We noticed that a few breast lesions had macro-calcifications, which may have been partially included in the ROI because the lesion was too small, which could have led to a higher HU value. Such errors can be corrected by calculating the ΔHU to reduce the partial volume effect. However, some malignant lesions have micro-calcifications (Fig 3), which are difficult to exclude when drawing the ROI. This may be the reason why the HU values on non-contrast-enhanced CT images higher than 32 HU had some power (AUC = 0.717) to detect malignancy.

In our study, the CT protocols were not optimized for breast pathology; instead, the study included routine chest CT and protocols to detect thoracic aneurysms or pulmonary emboli, which had scan times with an approximately 50-sec delay and a less than 30-sec delay, respectively. A study by Miyake et al. [14] used a protocol including a scan at 30 sec (early phase) and two minutes (delayed phase) after contrast injection; the authors noted that the best cut-off point for differentiating benign from malignant lesions was 60 HU in the early phase, which is similar to our result of 57 HU. Miyake et al. [14] also classified enhancement patterns into five types. Types 3, 4, and 5, which were all associated with rapid initial growth, had high PPVs for malignancy, with sustained, stable, and decreasing HU, respectively. These high PPVs may explain why—even though our patients did not undergo a dedicated breast CT protocol and the dynamic pattern was not available—the cut-off HU value was still clinically applicable; the most striking difference between malignant and benign lesions is the HU value in the early phase, not the delayed phase. The scanning time of our protocols resembled that of the early arterial phase.

Nineteen of the IBLs showed non-mass enhancement. Seventeen of them were proven to be malignant, and two were found to be fibrocystic changes. Baltzer [20] found that non-mass lesions were the major cause of false-positive magnetic resonance imaging (MRI) findings. In our study, the 17 malignant non-mass lesions all had values of more than 57 HU on contrast-enhanced CT and a ΔHU >33. In contrast, the two benign non-mass lesions both had enhancement lower than the cut-off values. Recognizing the enhancement pattern may be a reliable method for differentiating malignant and benign non-mass lesions. By paying attention to the breast in chest MDCT, non-mass lesions can be detected and treated at an earlier stage.

Our study had limitations. First, the assessment of morphology was subjective and had potential bias, as the readers had different experiences in reading breast images. Second, this was a retrospective study. There was still a possibility of selection bias, even though we recruited all patients who fulfilled the inclusion criterion. Third, the number of lesions (n = 102) included in our study was small. Randomized multicentre trials with larger sample sizes are needed to establish the relationship between CT enhancement values and malignancy. Fourth, pathological results were not available in all cases. Nevertheless, we trusted that imaging follow-up criteria were adequate to assume the benign nature of the breast lesions.

In conclusion, the addition of a quantitative CT enhancement value to the qualitative morphology of IBLs improves the diagnostic accuracy of the differentiation between benign and malignant lesions, even by less-experienced radiologists. We have shown that malignant lesions show significantly higher attenuation on contrast-enhanced CT in the early phase. Without a dedicated dynamic breast CT, we were still able to identify enhancing breast lesions and to use the cut-off value in combination with morphology to detect breast cancer in the early stage.

## Supporting Information

S1 Data Clinical data and CT measurements in this study. (XLSX)
